# Prognostic and Predictive Value of Systemic Inflammatory Markers in Epithelial Ovarian Cancer

**DOI:** 10.3390/medicina61030380

**Published:** 2025-02-22

**Authors:** Cem İdrisoğlu, Harun Muğlu, Jamshid Hamdard, Özgür Açıkgöz, Oktay Olmusçelik, Bahar Müezzinoğlu, Ömer Fatih Ölmez, Özcan Yıldız, Ahmet Bilici

**Affiliations:** 1 Department of Internal Medicine, Faculty of Medicine, Medipol University, Istanbul 34214, Turkey; cemidrisoglu@gmail.com (C.İ.); oolmuscelik@medipol.edu.tr (O.O.); 2 Department of Medical Oncology, Faculty of Medicine, Medipol University, Istanbul 34214, Turkey; jamshidhamdard@hotmail.com (J.H.); ozgur_acikgoz@yahoo.com (Ö.A.); omerfatih.olmez@medipol.com.tr (Ö.F.Ö.); ozcanyildiz71@gmail.com (Ö.Y.); ahmetknower@yahoo.com (A.B.); 3 Department of Medical Pathology, Faculty of Medicine, Medipol University, Istanbul 34214, Turkey; m.bahar@isnet.net.tr

**Keywords:** epithelial ovarian cancer, systemic inflammatory markers, prognostic factors, platinum resistance

## Abstract

*Background and Objectives:* Epithelial ovarian cancer (EOC) remains a significant global health challenge. While traditional prognostic factors are well established, emerging biomarkers continue to gain attention. *Materials and Methods:* This retrospective study evaluated the impact of systemic inflammatory markers on progression-free survival (PFS) and overall survival (OS) in 154 EOC patients. Pre-treatment neutrophil/lymphocyte ratio (NLR), platelet/lymphocyte ratio (PLR), and systemic inflammatory index (SII) were calculated and categorized into low and high groups. Univariate and multivariate analyses were conducted to identify independent prognostic factors, while logistic regression analysis was used to determine predictors of platinum resistance. *Results:* In the univariate analysis, elevated NLR and PLR were associated with poorer PFS and OS. However, these markers did not maintain statistical significance in the multivariate analysis. Although SII demonstrated a trend toward worse outcomes, it did not reach statistical significance. Histopathological type, PLR, and surgical approach were identified as independent predictors of platinum resistance. *Conclusions:* Our findings indicate that systemic inflammatory markers may hold prognostic value in EOC; however, further validation through larger prospective studies is necessary.

## 1. Introduction

Ovarian cancer remains a major global health concern, ranking as the second most common gynecologic malignancy in developed countries and the third in developing nations. Despite advancements in treatment, it continues to be the most lethal gynecologic cancer, with approximately 230,000 new cases diagnosed and 150,000 deaths reported worldwide each year [1,2].

Several factors have been recognized as key prognostic indicators in ovarian cancer, including age, residual tumor volume, performance status, and histological type. Additionally, recent studies have highlighted post-operative CA-125 (Cancer Antigen 125) levels, progesterone receptor status, and HER2 (human epidermal growth factor receptor 2) expression as important prognostic markers [3,4,5].

Ultrasonography is a cornerstone imaging modality in gynecologic oncology, offering noninvasive, real-time assessment of both uterine and ovarian pathologies. Among its various applications, B-mode ultrasound is widely utilized for detecting structural abnormalities, while Doppler ultrasound provides additional functional insights by evaluating vascular patterns [6]. The combination of these modalities has been shown to enhance diagnostic accuracy, particularly in distinguishing between benign and malignant uterine intracavitary pathologies (UIPs) in perimenopausal and postmenopausal women presenting with abnormal uterine bleeding (AUB). Notably, Doppler ultrasound enhances the ability to detect angiogenesis patterns—a hallmark of malignancy—improving sensitivity in differentiating between endometrial polyps, hyperplasia, fibroids, and endometrial carcinoma [6]. Given the accessibility and cost-effectiveness of ultrasonography, its optimization is particularly valuable in settings with limited resources where advanced imaging modalities such as MRI or CT are not always available. Similarly, Doppler ultrasound has been widely studied for its role in ovarian tumor assessment, as ovarian malignancies also exhibit characteristic vascular patterns that can aid in distinguishing benign from malignant ovarian lesions. Early detection of ovarian cancer remains challenging, and optimizing non-invasive diagnostic modalities, including Doppler ultrasonography, is crucial for improving clinical outcomes [7].

Ovarian cancer, the leading cause of gynecologic cancer-related mortality, is histopathologically classified into Type I and Type II epithelial ovarian cancers, which have distinct molecular profiles, clinical behaviors, and prognostic implications [8]. Type I ovarian cancers (low-grade serous, endometrioid, clear cell, mucinous, and malignant Brenner tumors) are typically slow-growing and diagnosed at earlier stages, while Type II tumors (high-grade serous carcinoma, carcinosarcoma, and undifferentiated carcinoma) exhibit aggressive behavior, high metastatic potential, and poorer survival outcomes [9,10]. Early and accurate classification is critical for personalized treatment strategies, yet conventional imaging techniques often fall short in differentiating these subtypes preoperatively [11].

Emerging ultrasound-based radiomics approaches offer a novel, quantitative framework for extracting high-throughput imaging features beyond what is visually perceptible to the human eye [12,13]. By applying machine learning algorithms to ultrasound data, radiomics can significantly improve diagnostic precision; as demonstrated in recent studies, where radiomics-based models achieved superior predictive performance compared to traditional imaging [14]. In particular, the integration of radiomics with clinical parameters—such as CA-125, HE4, and menopausal status—has been shown to enhance the ability to differentiate between Type I and Type II epithelial ovarian cancers, offering a noninvasive and clinically feasible alternative to histopathologic confirmation [14,15].

Tang et al. conducted a study investigating the role of ultrasound-based radiomics in the preoperative differentiation of Type I and Type II epithelial ovarian cancer. The study analyzed high-throughput imaging features extracted from ultrasound images and developed a predictive model to improve diagnostic accuracy. The results showed that the radiomics model achieved an AUC of 0.817 in the training set and 0.731 in the test set. When combined with clinical parameters such as CA-125, SCC, HE4, menopausal status, and ascites, the comprehensive model demonstrated superior performance, with an AUC of 0.982 in the training set and 0.886 in the test set. These findings suggest that ultrasound-based radiomics can serve as a valuable noninvasive tool for distinguishing epithelial ovarian cancer subtypes, contributing to more accurate diagnosis and personalized treatment planning in gynecologic oncology [9].

This retrospective study aimed to evaluate the impact of clinicopathological factors and pre-treatment systemic inflammatory markers on progression-free survival (PFS) and overall survival (OS) in patients with epithelial ovarian cancer (EOC). By analyzing these data, we sought to identify factors associated with patient survival and platinum resistance: a key determinant of treatment response and prognosis in ovarian cancer.

## 2. Materials and Methods

### 2.1. Study Design

This study initially screened 175 patients. Of these, 21 were excluded due to incomplete data (N = 7), loss to follow-up (N = 4), or a diagnosis of non-epithelial ovarian cancer (N = 10). The final cohort consisted of 154 patients (aged 21 to 88) who received neoadjuvant chemotherapy, underwent surgery, and/or received adjuvant chemotherapy. These patients were followed up at the Department of Oncology, Istanbul Medipol University, between 2013 and 2022.

### 2.2. Study Flowchart

**Figure 1:**
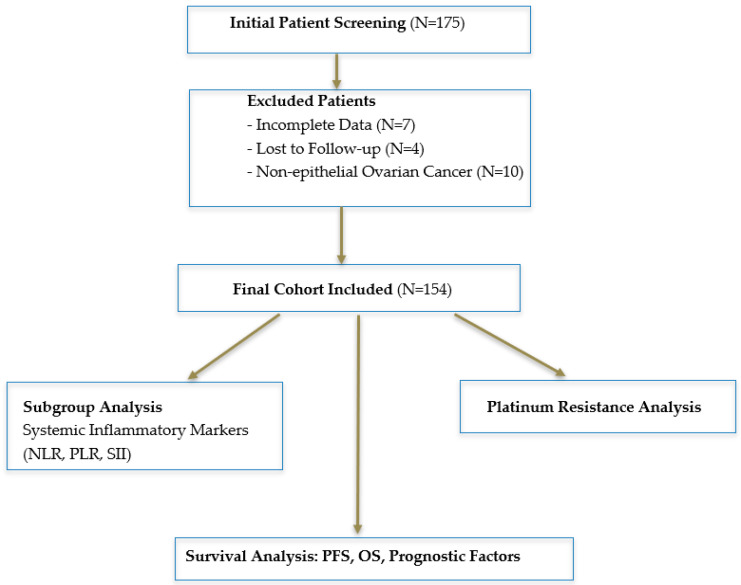
Study flowchart.

This flowchart illustrates the patient selection process, including inclusion and exclusion criteria for the study cohort (Figure 1). It provides a visual representation of how patients were screened, excluded, and included in the final analysis.

Patient staging was determined using the American Joint Committee on Cancer (AJCC) and Union for International Cancer Control (UICC) eighth edition, based on clinical and radiological findings at the time of initial diagnosis. Data collected included age at diagnosis, surgical details, FIGO (International Federation of Gynecology and Obstetrics) stage, ECOG PS (Eastern Cooperative Oncology Group Performance Status) scores, diagnosis and surgery dates, type of surgery, histological subtype, tumor localization, tumor grade, metastatic lymph nodes detected, number of excised lymph nodes, administration of neoadjuvant chemotherapy (NAC) and/or adjuvant chemotherapy (AC), NAC/AC regimen types, treatment dates, number of treatment cycles, CA-125 levels, BRCA status (if available), complete blood count (CBC) results before treatment, sites of relapse, surgical intervention at relapse (if performed), chemotherapy regimens used post-relapse, progression status and dates, and final patient status (alive or deceased).

Neutrophil-to-lymphocyte ratio (NLR), platelet-to-lymphocyte ratio (PLR), and systemic inflammatory index (SII) were calculated using pre-treatment neutrophil, lymphocyte, and platelet values from complete blood counts (CBCs). Their median cut-off values were then determined and used for categorization. The SII was calculated using the following formula: SII = (Platelet count × Neutrophil count)/Lymphocyte count. Patients were categorized into low and high groups for NLR (≤3 vs. >3), PLR (≤194 vs. >194), and SII (≤973.1 vs. >973.1), respectively. Among the 154 patients, 47.4% (*n* = 73) had low NLR (≤3), 46.8% (*n* = 72) had low PLR (≤194), and 44.9% (*n* = 69) had low SII (≤973.1).

Written informed consent was obtained from all participating patients or their designated relatives. This study was approved by the Local Ethics Committee of Medipol University (Istanbul, Turkey) on 5 May 2022 (decision number: 383). Patients with incomplete clinicopathological information were excluded from the study.

### 2.3. Baseline Characteristics of Patients

A total of 154 patients with EOC were included in this study, with a median age of 57 years. The majority of patients presented with advanced-stage disease and had good performance status (ECOG PS 0 or 1). Regarding tumor histology, serous papillary carcinoma was the most common subtype, accounting for 88.3% of cases. Most tumors were high-grade (89.6%). Due to advanced disease, 29.2% of patients received NAC prior to surgery. The majority underwent cytoreductive surgery, with 78.6% achieving maximal debulking. In terms of platinum sensitivity, 71.4% of patients were classified as platinum-sensitive (Table 1).

### 2.4. Statistical Analysis

All statistical analyses were performed using SPSS version 24.0 (SPSS Inc., Chicago, IL, USA). Descriptive statistics were reported as medians for continuous variables and within a 95% confidence interval (CI). Survival analyses and survival curves were generated using the Kaplan–Meier method, with comparisons conducted via log-rank tests.

PFS was defined as the time from the date of surgery or the date of response to medical treatment to either the first relapse/progression or, for non-relapsed cases, the last recorded follow-up visit. OS was defined as the time from diagnosis to either the last follow-up visit or the date of death.

Univariate and multivariate analyses, along with the Cox proportional hazards model, were used to assess survival outcomes in relation to clinicopathological features, NLR, PLR, and SII. Binary logistic regression analysis was applied to identify factors influencing platinum resistance. A 95% confidence interval (CI) was used to evaluate associations between OS and independent parameters. All *p*-values were two-sided, with statistical significance set at ≤0.05. The predictive performance of logistic regression models was assessed using receiver operating characteristic (ROC) curves, and the area under the curve (AUC) values were reported to evaluate discrimination ability.

## 3. Results

### Prognostic Factors and Survival Outcomes

The median PFS and OS were 19.6 months (95% CI: 17.0–22.2) and 59.6 months (95% CI: 49.2–69.6), respectively, with a median follow-up duration of 31.5 months.

Univariate analysis for PFS identified ECOG PS, primary tumor localization, surgical procedure, platinum resistance status, NLR, PLR, and SII as significant prognostic factors. Median PFS was significantly longer in platinum-sensitive patients compared to platinum-resistant patients (21.3 months vs. 9.3 months, *p* < 0.001).

**Figure 2:**
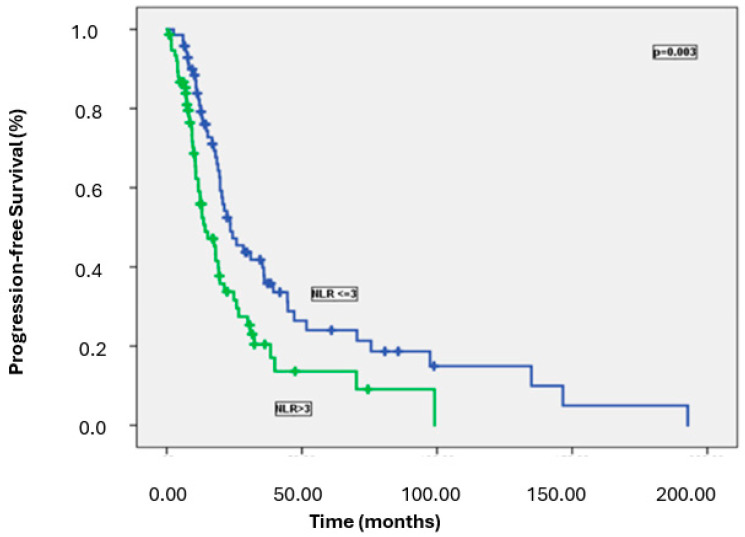
Progression-free survival rates according to NLR.

**Figure 3:**
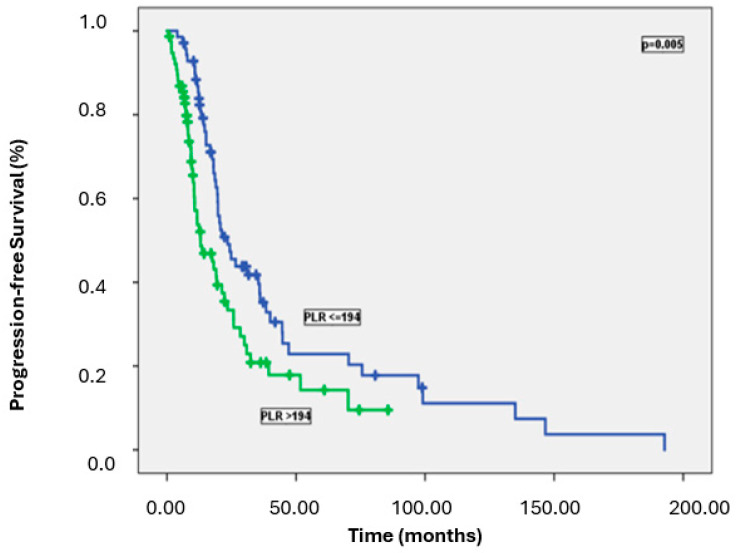
Progression-free survival curves according to PLR.

**Figure 4:**
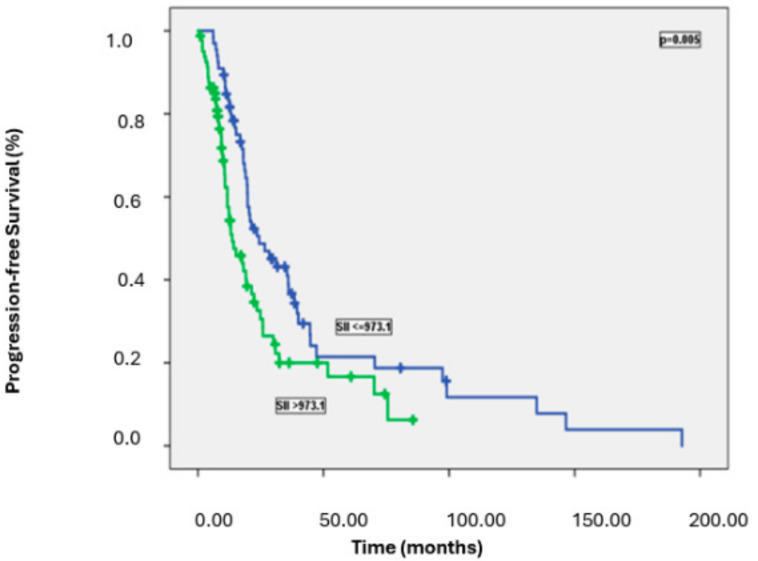
Progression-free survival curves according to Sll values.

Patients with high NLR, PLR, and SII had significantly worse PFS than those with lower levels. Specifically, the median PFS was 23.5 months in patients with low NLR versus 14.2 months in those with high NLR (*p* = 0.003); 23.5 months in patients with low PLR versus 13.0 months in those with high PLR (*p* = 0.005); and 24.3 months in patients with low SII versus 13.6 months in those with high SII (*p* = 0.005) (Figure 2, Figure 3 and Figure 4). Table 2 summarizes the results of the univariate analysis for PFS.

Univariate analysis for OS identified age, disease stage, ECOG PS, surgical procedure, platinum resistance status, NLR, and PLR as significant prognostic indicators. Median OS was significantly longer in patients with low NLR and PLR compared to those with high levels (61.1 months vs. 37.4 months, *p* = 0.02 and 64.4 months vs. 37.4 months, *p* = 0.008, respectively).

Although median OS was numerically higher in patients with SII ≤973.1 compared to those with SII >973.1 (60.7 months vs. 46.1 months), the difference did not reach statistical significance (*p* = 0.082). The results of the univariate analysis for OS are summarized in Table 3.

Multivariate analysis was performed to identify independent prognostic factors for PFS and OS based on significant variables from the univariate analysis. FIGO stage (*p* < 0.001, hazard ratio (HR): 1.89, 95% confidence interval (CI: 1.33–2.67), histopathological type (*p* = 0.006, HR: 1.69, 95% CI: 1.16–2.46), surgical type (*p* = 0.04, HR: 1.34, 95% CI: 0.96–1.88), and the presence of platinum resistance (*p* < 0.001, HR: 4.7, 95% CI: 2.42–9.12) were identified as independent prognostic factors for PFS (Table 2).

For OS, ECOG PS (*p* = 0.008, HR: 1.50, 95% CI: 1.11–2.02), surgical type (*p* = 0.045, HR: 1.62, 95% CI: 1.01–2.62), and platinum resistance (*p* = 0.004, HR: 3.02, 95% CI: 1.42–6.42) were found to be independent prognostic factors (Table 3). While univariate analysis demonstrated the prognostic impact of NLR, PLR, and SII on survival, their significance was not retained in the multivariate analysis.

Logistic regression analysis was conducted to assess predictors of platinum resistance; a key factor directly influencing survival. This analysis identified histopathological type (*p* = 0.004, OR: 4.06, 95% CI: 1.57–10.54), PLR (*p* = 0.03, OR: 4.08, 95% CI: 0.83–20.07), and surgical type (*p* = 0.032, OR: 1.84, 95% CI: 0.90–3.76) as independent predictors of platinum resistance (Table 4).

**Figure 5:**
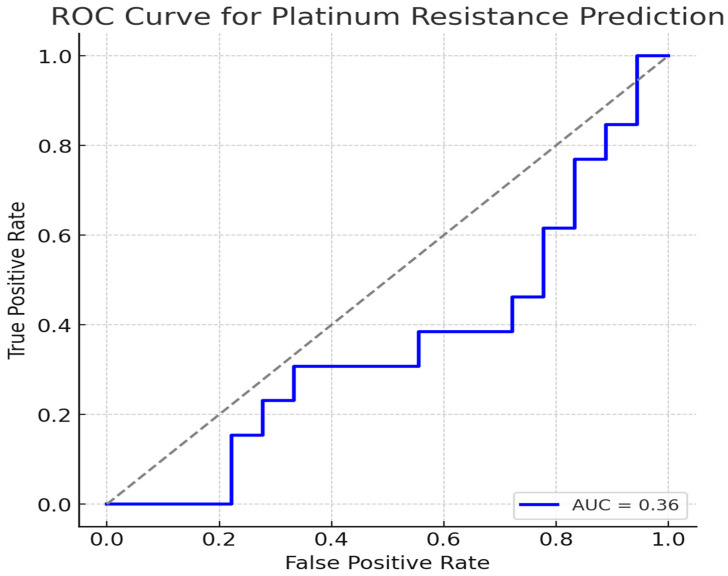
Receiver operating characteristic (ROC) curve for platinum resistance prediction.

The predictive performance of systemic inflammatory markers (NLR, PLR, SII), FIGO stage, ECOG PS, and surgical type for platinum resistance was evaluated using a logistic regression model. The receiver operating characteristic (ROC) curve was generated, and the area under the curve (AUC) was calculated. The AUC value was 0.36, indicating a limited predictive ability (Figure 5).

## 4. Discussion

Our study examined the prognostic and predictive value of systemic inflammatory markers in EOC. The key findings indicate that elevated NLR and PLR were significantly associated with poorer PFS and OS in univariate analysis. However, these markers lost statistical significance in multivariate analysis. The SII showed a trend toward worse outcomes but did not reach statistical significance. Moreover, we identified FIGO stage, histopathological type, surgical approach, and platinum resistance as independent prognostic factors for PFS; while ECOG PS, surgical approach, and platinum resistance were independently associated with OS. Additionally, histopathological type, PLR, and surgical type were independent predictors of platinum resistance.

Recent studies have demonstrated a strong link between inflammation and cancer. Elevated cytokine and chemokine levels have been implicated in tumor growth, angiogenesis, and metastasis. Emerging prognostic markers, such as NLR, PLR, and SII, derived from peripheral blood counts, serve as indicators of systemic inflammation and have shown prognostic significance in various solid tumors, including hepatocellular carcinoma, small cell lung carcinoma, and other lung cancers [16,17,18,19,20,21]. Our findings align with these studies, supporting the potential role of these markers in EOC prognosis.

Several meta-analyses have reported that high NLR and PLR values are associated with worse OS and PFS in ovarian cancer patients. Zhe Zhao et al. found that high NLR is linked to poorer OS and PFS in a meta-analysis including 13 studies and 3467 patients (HR: 1.70, 95% CI: 1.35–2.15 and HR: 1.77, 95% CI: 1.48–2.12, respectively) [22]. Similarly, Anastasia Prodromidou et al. highlighted NLR and PLR as promising prognostic factors in a meta-analysis of 18 studies involving 3453 ovarian cancer patients [23]. Another meta-analysis including 10 studies and 2019 ovarian cancer patients found that high NLR values were associated with worse OS (HR: 1.34, 95% CI: 1.16–1.54) and PFS (HR: 1.36, 95% CI: 1.17–1.57). Similarly, elevated PLR values were linked to worse OS (HR: 1.97, 95% CI: 1.61–2.40) and PFS (HR: 1.79, 95% CI: 1.46–2.20) [24].

On the other hand, Gatot Nyarumenteng et al. examined the impact of NLR and PLR on chemotherapy response in 116 ovarian cancer patients and found that those with high NLR (*p* = 0.026) and high PLR (*p* = 0.003) demonstrated better responses to chemotherapy [25].

Our study is consistent with previous research demonstrating that high NLR and PLR are associated with poorer outcomes. However, the lack of statistical significance in multivariate analysis may be due to the relatively small sample size and the heterogeneity of our patient population. Additionally, while our study did not establish a significant link between NAC and changes in NLR or PLR, this could be attributed to the limited number of patients receiving NAC.

Inflammation-related parameters have also been explored as early indicators of platinum resistance. Studies have shown that both NLR and PLR are associated with survival outcomes and response to chemotherapy [22,23,24,25]. For instance, M. Liontos et al. identified a significant association between high NLR and poor OS in ovarian cancer patients treated with NAC. While a decrease in NLR following NAC was predictive of a favorable response, the initial pre-NAC NLR level did not correlate with treatment response. However, patients with a high pre-NAC NLR who experienced a post-NAC decrease in NLR had better PFS compared to those whose NLR did not decrease [26,27].

The SII has also been investigated as a prognostic biomarker, particularly in ovarian and breast cancer. Research by Yongfang Ji and Haiyan Wang demonstrated a correlation between elevated SII levels and poorer OS in cervical, ovarian, and breast cancer. Additionally, higher SII levels have been associated with worse DFS and PFS in ovarian and triple-negative breast cancer [28]. A meta-analysis of 68 studies involving patients with peritoneal carcinomatosis following surgery confirmed the prognostic significance of SII, NLR, and PLR. High SII was identified as an independent risk factor for poor OS, while high PLR was associated with worse DFS in multivariate analysis. These findings suggest that SII, NLR, and PLR could serve as valuable prognostic markers in gynecologic and breast cancers.

Dan Nie et al.’s study demonstrated a strong correlation between high SII values and advanced disease stage, lymph node metastasis, and tumor recurrence in EOC. Moreover, high SII was significantly associated with poorer PFS and OS in both univariate and multivariate analyses [29].

The MITO24 study further investigated the prognostic significance of SII and NLR in EOC patients. High NLR was identified as an independent risk factor for 6-month PFS in patients treated with chemotherapy, but not in those receiving bevacizumab. Additionally, both high NLR and SII were independently associated with poorer OS in multivariate analysis, regardless of other clinical factors [30,31].

Recent studies have shown that a high peritoneal lavage fluid (PLF) ratio PLR is associated with poorer OS and PFS in ovarian cancer patients. Our study further supports this finding, demonstrating that a high PLR (>194) was significantly associated with worse PFS, while a low PLR (≤194) was linked to better OS in univariate analysis. However, in multivariate analysis, PLR was not identified as an independent prognostic factor [32,33,34].

Our study confirmed the prognostic significance of well-established factors such as FIGO stage, ECOG PS, surgical type, and platinum sensitivity. Additionally, we identified histopathological type, PLR, and surgical type as independent predictors of platinum resistance, aligning with prior research suggesting that these factors influence treatment response.

This study has certain limitations, including its retrospective design, relatively small sample size, and short follow-up period. However, its strength lies in its focus on the predictive value of PLR, NLR, and SII for platinum sensitivity, providing novel insights into the literature. Future prospective studies with larger and more diverse patient cohorts are needed to validate these findings and further explore the clinical utility of systemic inflammatory markers in EOC prognosis.

In conclusion, our study highlights the prognostic significance of systemic inflammatory markers in ovarian cancer while reinforcing the importance of established prognostic factors. Although these markers were associated with poorer outcomes in univariate analysis, their independent prognostic value remains uncertain. Further research is required to assess their role in risk stratification and personalized treatment strategies for patients with EOC.

## 5. Conclusions

This retrospective study underscores the prognostic significance of systemic inflammatory markers, including NLR, PLR, and SII, in patients with EOC. Elevated levels of these markers were associated with poorer PFS and OS, reinforcing their potential role as prognostic indicators. While well-established factors such as FIGO stage, ECOG PS, surgical type, and platinum sensitivity remain critical in prognosis, integrating inflammatory markers into clinical evaluation may provide additional prognostic insights.

Furthermore, our study identified histopathological type, PLR, and surgical type as independent predictors of platinum resistance, emphasizing the importance of these factors in treatment planning and therapeutic decision-making for ovarian cancer patients. Although some associations were observed, the prognostic significance of inflammatory markers was weaker in multivariate analysis, likely due to the study’s limited sample size and patient heterogeneity.

To establish the clinical utility of these markers in predicting treatment response and guiding individualized treatment strategies, further prospective studies with larger and more diverse patient cohorts are warranted. Validating these findings in future research may enhance risk stratification and optimize treatment approaches for EOC patients.

## Figures and Tables

**Table 1:** Clinicopathological characteristics of patients.

Characteristic	*n* (%)
Age, year	
Interval	21–88
Median	57
Stage	
Stage I	21(13.6)
Stage II	6 (3.9)
Stage III	111 (72.1)
Stage IV	16 (10.4)
Eastern Cooperative Oncology Group Performance Status	
0	94 (61.0)
I	41 (26.6)
II	13 (8.4)
III	6 (3.9)
Primary Localization	
Ovary	127 (82.5)
Fallopian tube	18 (11.7)
Primary peritoneal	9 (5.8)
Histopatological type	
Serous papillary	136 (88.3)
Clear cell	10 (6.5)
Mucinous	6 (3.9)
Borderline	2 (1.3)
Grade	
Low	8 (5.2)
High	138 (89.6)
Unknown	8 (5.2)
NAC	
Yes	45 (29.2)
No	109 (70.8)
Surgical Type	
Maximal debulking	121 (78.6)
Optimal debulking	20 (13.0)
Suboptimal debulking	4 (2.6)
Inoperable	3 (1.9)
Adjuvant Chemotherapy	
Yes	143 (92.9)
No	11 (7.1)
Relapse Status	
Yes	105 (68.1)
No	49 (31.9)
Platinum Resistance	
Partially sensitive	11 (7.2)
Sensitive	110 (71.4)
Resistant	33 (21.4)

NAC: Neoadjuvant chemotherapy. Categorical variables were analyzed using the chi-square test, and continuous variables were analyzed using the Mann–Whitney U test.

**Table 2:** The results of univariate and multivariate analysis for PFS.

Characteristics	*n* (%)	Median PFS (Month)	Univariate *p*-Value	HR CI 95%	Multivariate *p*-Value
Age, year			0.64		
<60	86 (55.8)	19.5			
≥60	68 (44.2)	19.7			
Stage			0.015	1.89 1.33–2.67	<0.001
Stage I	21(13.6)	51.7			
Stage II	6 (3.9)	17.5			
Stage III	111 (72.1)	19.2			
Stage IV	16 (10.4)	13.0			
ECOG PS			0.029	1.15 0.89–1.48	0.27
0-I	135 (87.6)	21.2			
II-III	19 (22.4)	18.0			
Primary Localization			0.024	1.44 0.90–2.31	0.11
Ovary	127 (82.5)	19.7			
Fallopian tube	18 (11.7)	13.5			
Primary peritoneal	9 (5.8)	NR			
Histopatological Type			0.09	1.69 1.16–2.46	0.006
Serous papillary	136 (88.3)	20.3			
Clear cell	10 (6.5)	6.9			
Mucinous	6 (3.9)	6.2			
Borderline	2 (1.3)	12.6			
Grade			0.42		
Low	8 (5.2)	NR			
High	138 (89.6)	19.7			
Unknown	8 (5.2)	NR			
NAC			0.54		
Yes	45 (29.2)	19.0			
No	109 (70.8)	20.3			
Surgical Type			0.003	1.34 0.96–1.88	0.04
Maximal debulking	121 (78.6)	21.2			
Optimal debulking	20 (13.0)	19.5			
Suboptimal debulking	4 (2.6)	13.3			
Inoperable	3 (1.9)	7.4			
Platinum Resistance			<0.001	4.70 2.42–9.12	<0.001
Sensitive	121 (78.6)	21.3			
Resistant	33 (21.4)	9.3			
NLR			0.003	1.53 0.81–2.91	0.18
≤3	73 (47.4)	23.5			
>3	81 (52.6)	14.2			
PLR			0.005	0.87 0.46–1.64	0.67
≤194	72 (46.8)	23.5			
>194	82 (53.2)	13.0			
SII			0.005	1.67 0.79–3.53	0.17
≥973.1	69 (44.9)	24.3			
>973.1	85 (55.1)	13.6			

PFS: progression-free survival, NA: not available, NR: not reached, NLR: neutrophil/lymphocyte ratio, SII: systemic inflammatory index, ECOG PS: Eastern Cooperative Oncology Group Performance Status, HR: hazard ratio. The Kaplan–Meier method and log-rank test were used for univariate analysis, and the Cox regression model was used for multivariate analysis. All *p*-values were two-sided, with statistical significance set at ≤0.05.

**Table 3:** Univariate and multivariate analysis for OS.

Characteristics	*n* (%)	Median OS (Month)	Univariate *p*-Value	HR CI 95%	Multivariate *p*-Value
Age, Year			0.50		
<60	86 (55.8)	57.1			
≥60	68 (44.2)	55.8			
Stage			0.04	1.33 0.82–2.15	0.23
Stage I	21(13.6)	NR			
Stage II	6 (3.9)	NR			
Stage III	111 (72.1)	51.7			
Stage IV	16 (10.4)	29.1			
ECOG PS			<0.001	1.50 1.11–2.02	0.008
0-I	135 (87.6)	62.0			
II-III	19 (22.4)	29.1			
Primary Localization			0.90		
Ovary	127 (82.5)	NR			
Fallopian tube	18 (11.7)	NR			
Primary peritoneal	9 (5.8)	NR			
Histopatological Type			0.48		
Serous papillary	136 (88.3)	51.7			
Clear cell	10 (6.5)	NR			
Mucinous	6 (3.9)	NR			
Borderline	2 (1.3)	NA			
Grade			0.30		
Low	8 (5.2)	NR			
High	138 (89.6)	57.1			
Unknown	8 (5.2)	NR			
NAC			0.30		
Yes	45 (29.2)	38.5			
No	109 (70.8)	56.9			
Surgical Type			0.001	1.62 1.01–2.62	0.045
Maximal debulking	121 (78.6)	62.0			
Optimal debulking	20 (13.0)	56.9			
Suboptimal debulking	4 (2.6)	45.5			
Inoperable	3 (1.9)	10.4			
Platinum Resistance			<0.001	3.02 1.42–6.42	0.004
Sensitive	121 (78.6)	64.6			
Resistant	33 (21.4)	25.8			
NLR			0.02	1.08 0.42–2.80	0.87
≤3	73 (47.4)	61.1			
<3	81 (52.6)	37.4			
PLR			0.008	1.46 0.59–3.61	0.40
≤194	72 (46.8)	64.6			
>194	82 (53.2)	37.4			
SII			0.082	1.08 0.34–3.34	0.89
≥973.1	69 (44.9)	60.7			
>973.1	85 (55.1)	46.1			

OS: overall survival, NA: not available, NR: not reached, NLR: neutrophil/lymphocyte ratio, PLR: platelet/lymphocyte ratio, SII: systemic inflammatory index, ECOG PS: Eastern Cooperative Oncology Group Performance Status, HR: hazard ratio. The Kaplan–Meier method and log-rank test were used for univariate analysis, and the Cox regression model was used for multivariate analysis. All *p*-values were two-sided, with statistical significance set at ≤0.05.

**Table 4:** The analysis of predictors of platinum resistance.

Factors	β	X^2^	*p*	OR	95% CI
FIGO stage	0.20	0.21	0.64	1.22	0.51–2.89
EGOG PS	0.24	0.09	0.75	1.28	0.27–5.99
Histopathology	1.40	8.35	0.004	4.06	1.57–10.54
Surgical type	0.61	2.84	0.032	1.84	0.90–3.76
NLR	−0.27	0.08	0.77	0.75	0.11–5.14
PLR	1.40	2.99	0.03	4.08	0.83–20.07
SII	−1.42	1.48	0.22	0.24	0.02–2.37

OR: relative risk for platinum resistance, CI: confidence interval, NLR: neutrophil/lymphocyte ratio, PLR: platelet/lymphocyte ratio, SII: systemic inflammatory index. FIGO: International Federation of Gynecology and Obstetrics. Logistic regression analysis was used to evaluate predictors of platinum resistance. All *p*-values were two-sided, with statistical significance set at ≤0.05.

## Data Availability

The original contributions presented in this study are included in the article. Further inquiries can be directed to the corresponding author.
